# Ten-Year Atherosclerotic Cardiovascular Disease Risk in Metabolic Dysfunction-Associated Steatotic Liver Disease (MASLD): Separate Analyses from Romanian and Italian Cohorts Integrating Metabolic, Hepatic, and Gut–Liver Axis Markers

**DOI:** 10.3390/jcm14238361

**Published:** 2025-11-25

**Authors:** Naomi-Adina Ciurea, Cristina Monica Pantea, Paul Grama, Irina-Bianca Kosovski, Ilaria Farella, Simona Bataga, Agostino Di Ciaula, Piero Portincasa

**Affiliations:** 1 Department of Internal Medicine, “George Emil Palade” University of Medicine, Pharmacy, Science and Technology of Targu Mures, 540139 Targu Mures, Romania; paul.grama@umfst.ro (P.G.); simonabataga@yahoo.com (S.B.); 2 Doctoral School of Medicine and Pharmacy, “George Emil Palade” University of Medicine, Pharmacy, Sciences and Technology of Targu Mures, 38 Gheorghe Marinescu Street, 540139 Targu Mures, Romania; 3 Doctoral School of Public Health, Clinical Medicine and Oncology, University of Bari “Aldo Moro”, 70121 Bari, Italy; 4 Department of Clinical Science-Internal Medicine, “George Emil Palade” University of Medicine, Pharmacy, Science and Technology of Targu Mures, 540139 Targu Mures, Romania; cmonicapantea@gmail.com; 5 Department of Pathophysiology, “George Emil Palade” University of Medicine, Pharmacy, Science and Technology of Targu Mures, 540139 Targu Mures, Romania; bianca.kosovski@umfst.ro; 6 Department of Medicine and Surgery, LUM University, 70010 Casamassima, Italy; ilafarella@yahoo.com; 7 Clinica Medica “Augusto Murri”, Department of Precision and Regenerative Medicine and Ionian Area (DiMePre-J), University of Bari “Aldo Moro”, 70121 Bari, Italy; agodiciaula@gmail.com (A.D.C.); piero.portincasa@uniba.it (P.P.)

**Keywords:** MASLD, ASCVD, cardiovascular risk, liver fibrosis, intestinal permeability, zonulin, lactulose/mannitol, hypertension, diabetes, ARFI, FibroTest

## Abstract

**Background/Objectives**: Metabolic dysfunction–associated steatotic liver disease (MASLD) is closely linked to atherosclerotic cardiovascular disease (ASCVD), but the prognostic value of liver fibrosis and gut–liver axis alterations remains uncertain. **Methods**: We conducted a prospective, observational study in two tertiary centers (in Romania and Italy) and compared the outcomes with different tests available for fibrosis (FibroTest in Romania or acoustic radiation force impulse (ARFI) elastography in Italy) and intestinal permeability (IP) (by fecal zonulin in Romania or lactulose/mannitol ratio in Italy). Liver steatosis was confirmed at ultrasonography. Analyses followed a within-cohort strategy. Ten-year ASCVD categories were summarized separately per cohort, and within-cohort associations with elevated ASCVD risk (≥7.5%) were explored using univariate logistic regression with age-adjusted two-parameter checks. A pooled robustness analysis (*n* = 132) was then performed using multivariable logistic regression models for intermediate–high ASCVD risk (≥7.5%), adjusted for age (per 5 years), waist circumference (per 5 cm), total cholesterol (per 10 mg/dL), diabetes, and hypertension. A higher threshold (≥20%) yielded the same qualitative interpretation. **Results**: ASCVD was computable for 52 Romanian (low 78.8%, borderline 5.8%, intermediate 7.7%, high 7.7%) and 80 Italian participants (low 80.0%, borderline 6.2%, intermediate 12.5%, high 1.2%). In both cohorts, age was associated with higher ASCVD. Fibrosis severity (FibroTest or ARFI) and IP (zonulin or LA/MA) showed no associations with ASCVD. In pooled adjusted models, neither significant fibrosis nor high intestinal permeability was independently associated with ASCVD, whereas age and cardiometabolic comorbidities remained the dominant correlates. **Conclusions**: Across both cohorts, 10-year ASCVD risk was mainly determined by age and major cardiometabolic comorbidities. Neither liver fibrosis nor intestinal permeability contributed additional prognostic value in this setting, regardless of the assessment method. These data support prioritizing aggressive metabolic risk management and call for harmonized, longitudinal studies to clarify gut–liver contributions to cardiovascular outcomes.

## 1. Introduction

Metabolic dysfunction-associated steatotic liver disease (MASLD) has emerged as a major global health concern, mirroring the escalating prevalence of obesity, insulin resistance, and type 2 diabetes across diverse populations worldwide [1]. Recent epidemiological studies estimate that MASLD affects approximately 25–30% of the global adult population, with increasing prevalence observed in Europe, particularly in Southern and Eastern European countries [1]. In 2023, an international consensus panel adopted the MASLD terminology to better capture the metabolic drivers of this condition and align clinical practice with contemporary understanding of its pathophysiology [2]. Beyond being a hepatic disorder, MASLD is now recognized with wide-ranging consequences, most notably an increased risk of cardiovascular complications, which represent the leading cause of death among affected individuals [3,4]. Cardiovascular mortality in MASLD has been reported to be two-to four-fold higher compared to individuals without liver involvement, highlighting the urgent need for early risk identification [5].

While early stages of hepatic steatosis are often asymptomatic, progressive liver damage and systemic metabolic disturbances significantly increase the likelihood of adverse outcomes. Among these, cardiovascular disease (CVD) is of particular concern, given its high prevalence and its role as the primary determinant of long-term morbidity and mortality in MASLD [6,7]. Traditional cardiometabolic risk factors, such as central obesity, dyslipidemia, and hyperglycemia, interact with hepatic inflammation and fibrosis to amplify vascular injury [3,8]. Consequently, precise and standardized tools for cardiovascular risk assessment are essential. The atherosclerotic cardiovascular disease (ASCVD) score is widely used and validated for stratifying cardiovascular risk in clinical and research settings [9,10].

Several pathways have been proposed to explain the relationship between MASLD and cardiovascular disease. In addition to classical metabolic parameters, the gut–liver axis has attracted growing interest as a potential driver of systemic inflammation and vascular dysfunction [11,12]. Altered intestinal permeability facilitates the translocation of microbial products, triggering hepatic and systemic immune responses that may contribute to both progression of liver disease and cardiovascular complications [13,14]. However, data linking gut barrier dysfunction to cardiovascular outcomes remain limited and inconsistent, underscoring the need for studies integrating gut–liver axis markers with metabolic and hepatic parameters. Non-invasive biomarkers, such as fecal zonulin [15], and functional tests, including sugar absorption assays, offer complementary approaches to assess gut barrier integrity [16]. Similarly, accurate characterization of liver fibrosis—through tools such as FibroMax [17] or acoustic radiation force impulse (ARFI) elastography [18]—provides critical information for risk stratification, as fibrosis remains the strongest predictor of hepatic and extrahepatic outcomes [19].

Although the links between MASLD, liver fibrosis, and cardiovascular disease are well established, most studies have been limited to single cohorts using different diagnostic techniques. Few investigations have attempted to integrate metabolic, hepatic, and gut–liver axis parameters to comprehensively evaluate predictors of cardiovascular risk comparing different approaches of assessment. Furthermore, there is limited evidence on how these relationships manifest across different geographical clinical settings, where diagnostic techniques may vary.

The aim of the present study was, therefore, to measure ASCVD in two independent European cohorts of subjects with MASLD and to assess the role of IP and liver fibrosis, assessed with different techniques, in modulating the individual cardiovascular risk [20,21].

## 2. Materials and Methods

### 2.1. Study Design and Population

This was a prospective, observational, multicenter cohort study conducted in two independent MASLD cohorts enrolled in Romania or Italy. Overall, 52 participants in Romania and 80 participants in Italy were enrolled, each with complete baseline, fibrosis, intestinal permeability, and ASCVD assessment. In both cohorts, eligibility criteria included age ≥18 years, a Body Mass Index (BMI) ranging from 18.5 and 40 kg/m^2^, and evidence of hepatic steatosis confirmed through imaging (abdominal ultrasound), non-invasive diagnostic algorithms, or liver biopsy. Exclusion criteria were excessive alcohol consumption (>20 g/day for women and >30 g/day for men), lack of informed consent, pregnancy or lactation, cirrhosis of toxic or alcoholic etiology, history of bariatric surgery of current surgical candidacy, severe psychiatric disorders, advanced cardiovascular disease, and renal insufficiency.

### 2.2. Ethical Approval

The study protocol was reviewed and approved by the Ethics Committee of the County Clinical Emergency Hospital of Targu Mures, Romania (Approval No. Ad. 5004/16.02.2023), and by the Ethics Committee of the Department of Medicine, University of Bari, Italy (Study No. 65, Protocol No. 62806; FUEPEN). Written informed consent was obtained from all participants prior to study enrollment. All procedures were performed in accordance with the ethical principles outlined in the Declaration of Helsinki.

### 2.3. Clinical and Laboratory Assessments

At baseline, all participants underwent a comprehensive clinical and laboratory evaluation. Anthropometric measurements included weight, height, BMI, and waist circumference. BMI was calculated as weight (kg) divided by height squared (m^2^) and categorized according to World Health Organization criteria. Waist circumference was measured at the midpoint between the lowest rib and the iliac crest using a non-stretchable tape, following NCEP-ATP III recommendations. Blood pressure was measured in a seated position after a minimum of five minutes of rest, using a calibrated sphygmomanometer.

### 2.4. Operational Definitions and Pre-Analytical Handling

Hypertension was defined as a prior medical diagnosis, use of antihypertensive medication, or an average seated systolic blood pressure ≥ 140 mmHg and/or diastolic ≥ 90 mmHg on two measurements during the visit. Diabetes mellitus was defined by prior diagnosis, use of glucose-lowering therapy, fasting plasma glucose ≥ 126 mg/dL, or HbA1c ≥ 6.5% when available. Smoking status was categorized as current, former, or never. Alcohol consumption was recorded as any self-reported intake in the past 12 months. Fasting venous blood samples were collected to measure plasma glucose, insulin, total cholesterol, triglycerides, alanine aminotransferase (ALT), aspartate aminotransferase (AST), and gamma-glutamyl transferase (GGT). Insulin resistance was estimated using the homeostasis model assessment of insulin resistance (HOMA-IR), calculated by the formula: fasting insulin (µU/mL) × fasting glucose (mmol/L) ÷ 22.5.

### 2.5. Cardiovascular Risk Assessment

In both cohorts, the cardiovascular risk was calculated using the atherosclerotic cardiovascular disease (ASCVD) risk score, based on the American College of Cardiology/American Heart Association (ACC/AHA) guidelines [22]. The ASCVD score incorporates key variables including age, sex, total cholesterol, high-density lipoprotein (HDL) cholesterol, systolic blood pressure, diabetes status, and smoking history. Risk was categorized as low (<5%), borderline (5–7.4%), intermediate (7.5–19.9%), or high (≥20%).

### 2.6. Assessment of Liver Fibrosis

Different diagnostic modalities were applied in the Romanian and Italian cohorts, according to the standard practices at each clinical center and availability of instruments.

In the Romanian cohort, liver fibrosis was assessed using the FibroMax algorithm (BioPredictive, Paris, France), a validated panel of serum biomarkers that integrates multiple biochemical parameters to stage hepatic fibrosis and steatosis. FibroTest, a component of the FibroMax panel, was computed from α2-macroglobulin, apolipoprotein A1, haptoglobin, total bilirubin, and gamma-glutamyl transferase (GGT), automatically adjusted for age and sex using the manufacturer’s algorithm. Internal quality control flags (e.g., hemolysis, Gilbert syndrome) were checked before accepting each result.

In the Italian cohort, liver fibrosis was assessed by ARFI, according to a standardized procedure. Briefly, for each patient, ≥10 valid stiffness measurements were obtained from the right liver lobe via intercostal spaces during quiet respiration. Results were expressed in meters per second (m/s) and classified into fibrosis stages (F0–F4) according to locally validated MASLD cut-offs. Only exams with an interquartile range/median (IQR/M) ≤ 30% were included for analysis.

In the two cohorts, significant fibrosis was defined as ≥F2 based on the local method (Romania: FibroTest; Italy: ARFI).

### 2.7. Assessment of Intestinal Permeability (IP)

In the Romanian cohort, IP was evaluated by quantifying fecal zonulin concentrations using a commercially available enzyme-linked immunosorbent assay (ELISA) kit (Immundiagnostik AG, Bensheim, Germany), following the manufacturer’s instructions. Stool samples were collected by participants within 24 h prior to the visit using sterile, leak-proof containers. Samples were stored at 2–8 °C during transport and delivered to the laboratory on cold packs. Upon receipt, homogenized aliquots were frozen at −20 °C until batch analysis. Each ELISA plate included blank wells and two-level control samples, and all patient samples were run in duplicate. The assay had a lower detection limit of 5 ng/mL, and values > 107 ng/mL were considered indicative of increased intestinal permeability, based on the reference range provided by the manufacturer. Inter- and intra-assay coefficients of variation were maintained below 10%. Elevated intestinal permeability was predefined as fecal zonulin > 107 ng/mL.

In the Italian cohort, IP were assessed using a sugar absorption test. Patients ingested a standardized solution containing lactulose and mannitol after an overnight fast. The test solution consisted of 5 g lactulose and 2 g mannitol dissolved in 100 mL of water. Participants were instructed to void their bladder before ingestion, then to collect all urine over the subsequent 5 h into containers containing preservative. Total urine was recorded and aliquots were frozen at −20 °C until analysis. Sugar concentrations were analyzed using high-performance liquid chromatography (HPLC) (Agilent Technologies, Santa Clara, CA, USA). The lactulose/mannitol (LA/MA) ratio was subsequently calculated, with values > 0.03 indicating increased intestinal permeability [23,24]. Elevated intestinal permeability was defined as LA/MA ratio > 0.03 (Italy).

### 2.8. Questionnaires and Patient-Reported Outcomes

Participants completed a structured set of validated self-report questionnaires to assess lifestyle and psychosocial factors. Dietary adherence was evaluated using the Mediterranean Diet Score (MEDScore) [25]. Health-related quality of life was measured using the 36-Item Short Form Health Survey (SF-36) [26]. Depressive symptoms were screened using the Patient Health Questionnaire-9 (PHQ-9) [27]. Additional instruments assessed stress perception and behavioral patterns relevant to the gut–liver axis. Questionnaires were administered under standardized conditions by trained personnel. Questionnaire packets used validated language versions (Romanian and Italian) where available. The Mediterranean Diet adherence was quantified using the MEDScore (0–14 points). SF-36 domain scores were transformed to 0–100 scales; higher scores indicate better health status. PHQ-9 total scores (0–27) were categorized as minimal (0–4), mild (5–9), moderate (10–14), moderately severe (15–19), and severe (20–27) for descriptive purposes.

### 2.9. Statistical Analysis

Analyses were performed within each cohort (Romania or Italy) using NCSS v10 (NCSS, LLC, Kaysville, UT, USA). The primary outcome was the distribution of 10-year ASCVD risk categories (<5%, 5.0–7.4%, 7.5–19.9%, ≥20%) summarized separately for each cohort. An exploratory, descriptive comparison of ASCVD category distribution between Romania and Italy was prespecified; no other cross-cohort statistical testing was undertaken. The secondary objective assessed within-cohort associations of grade liver fibrosis and IP with ASCVD risk. Because events were limited, we prespecified a binary outcome of ASCVD ≥ 7.5% (intermediate/high) vs. <7.5% to ensure reasonable events per parameter. Univariate logistic regressions provided odds ratios (OR) and 95% confidence intervals (CI) for demographic, anthropometric and biochemical variables. Given the exploratory scope and few events, no multivariable models were fit; instead, gut–liver axis and fibrosis markers were additionally examined in age-adjusted two-parameter checks. As sensitivity analyses, the binary outcome ASCVD ≥ 20% (high risk) was evaluated using Fisher’s exact tests for dichotomous exposures (significant fibrosis, elevated intestinal permeability). Where a 0 cell occurred, the Haldane–Anscombe correction was applied for OR estimation, and exact two-sided *p*-values are reported. To preserve information from continuous measures, we computed Spearman correlations between ASCVD% and (i) intestinal permeability and (ii) fibrosis stage, within each cohort. All tests were two-sided with α = 0.05. No multiplicity adjustments were applied, consistent with the exploratory nature of the study.

Additionally, the two cohorts were pooled (*n* = 132) and multivariable logistic regression models were fitted for intermediate–high ASCVD risk (≥7.5%). Two exposures of interest were evaluated: significant liver fibrosis (≥F2) and high intestinal permeability, defined as fecal zonulin > 107 ng/mL in the Romanian cohort or LA/MA ratio > 0.03 in the Italian cohort. Models were adjusted for age (per 5 years), waist circumference (per 5 cm), total cholesterol (per 10 mg/dL), diabetes, and hypertension. Analyses using a higher threshold (ASCVD ≥ 20%) provided concordant qualitative results.

Given the heterogeneity of diagnostic tools used in the two cohorts, multivariable analyses were not performed separately within each center to avoid instability generated by small strata. The pooled multivariable logistic regression model was therefore considered exploratory, and its interpretation was carried out with caution. Variables were selected based on biological plausibility and absence of multicollinearity, acknowledging that fibrosis and permeability markers were measured through different but validated modalities in the two settings.

## 3. Results

### 3.1. Baseline Characteristics

A total of 132 adults with MASLD were enrolled: 52 in the Romanian cohort and 80 in the Italian cohort. Baseline characteristics in the two cohorts are summarized in Table 1. Overall, both samples comprised middle-aged adults with anthropometric profiles consistent with overweight to mild obesity and laboratory values compatible with a cardiometabolic-risk population. The Romanian and the Italian cohort exhibited broadly comparable distributions of age, sex, and insulin resistance indices, with some variations in lipid profile and blood pressure values.

A complete raw data summary for both cohorts is provided in Supplementary Table S1.

### 3.2. Ten-Year ASCVD Risk Distribution by Cohort

Table 2 summarizes the 10-year ASCVD risk distribution within each cohort (Romania, *n* = 52; Italy, *n* = 80) and reports between-cohort *p*-values for each category.

There was no statistically significant difference in the overall distribution of ASCVD categories between cohorts (Chi-square test, *p* > 0.05). At the category level, no individual category differed significantly between Romania and Italy (Fisher’s exact tests, all *p* > 0.05). A higher proportion classified as high risk (≥20%) in Romania vs. Italy (7.7% vs. 1.2%) was observed but did not reach statistical significance.

### 3.3. Liver Fibrosis

FibroTest staging is detailed in Table 3. Stages clustered in F0–F1 (75% and 82.5%), while significant fibrosis (F2–F4), occurred in 3/52 subjects (5.8%) and in 17.5% of Romanian and Italian patients, respectively (*p* = ns).

### 3.4. Intestinal Permeability

Table 4 shows that in the Romanian cohort, median fecal zonulin concentration was 56.5 [37.5–107.3] ng/mL. Concentration exceeded the predefined threshold (>107 ng/mL) in 14 subjects (26.9%) (Table 4).

In the Italian cohort, the median LA/MA ratio was 0.014 [0.011–0.017], with 5 subjects (6.3%) exceeding the predefined cut-off (>0.03). This difference was significant different according to Chi-squared Fisher’s Exact test (*p* = 0.002).

### 3.5. Within-Cohort Predictors of Elevated ASCVD Risk

Elevated 10-year risk was prespecified as ASCVD ≥ 7.5% (intermediate or high) versus <7.5% to ensure adequate events per parameter. All analyses were performed separately by cohort and are exploratory. Odds ratios (OR) with 95% confidence intervals (CI) from univariate logistic regression are summarized in Table 5A,B. Given the small number of events, multivariable models were not attempted; instead, IP and liver fibrosis were additionally examined in age-adjusted two-parameter models.

As depicted in Table 5A, in the Romanian cohort, age was associated with higher odds of elevated risk (OR per 5 years 1.65; 95% CI 1.17–2.34). Hypertension (21.00; 2.89–152.58) and diabetes (5.00; 1.02–24.41) were also strongly correlated. Total cholesterol was positively associated (per 10 mg/dL 1.26; 1.01–1.56), whereas BMI, waist circumference, HDL-C, smoking, and sex yielded imprecise estimates. IP and liver fibrosis were not associated with ASCVD: zonulin > 107 ng/mL 1.80 (0.37–8.79) and significant fibrosis (≥F2 by FibroTest) 3.00 (0.24–37.67). Results were confirmed when models were adjusted for age (zonulin > 107 ng/mL 1.78; 0.26–12.16; significant fibrosis 0.31; 0.02–5.57).

In the Italian cohort (Table 5B), as in the Romanian cohort, age was strongly associated with elevated cardiovascular risk (OR per 5 years 2.21; 1.41–3.45). Waist circumference was also positively associated with ASCVD risk (per 5 cm 1.38; 1.01–1.88). Fasting glucose showed a borderline association (per 10 mg/dL 1.47; 1.00–2.17) and HDL-C a borderline inverse association (per 5 mg/dL 0.78; 0.61–1.00). Hypertension and diabetes pointed toward a higher risk, but with wide CIs. Neither significant fibrosis (≥F2 by ARFI) (1.33; 0.26–6.94) nor elevated intestinal permeability (LA/MA > 0.03) showed evidence of association. Notably, no ASCVD ≥ 7.5% events occurred among LA/MA-positive participants (two-sided Fisher’s exact *p* = 1.00).

### 3.6. Sensitivity and Robustness Analyses

In the pooled dataset (*n* = 132, i.e., 52 from the Romanian cohort and 80 from the Italian cohort), we fitted separate multivariable logistic regression models to estimate adjusted odds ratios (aORs) for intermediate–high 10-year ASCVD risk (≥7.5%) associated with either significant liver fibrosis (≥F2) or high intestinal permeability. For intestinal permeability, a harmonized definition was applied: fecal zonulin >107 ng/mL in the Romanian cohort and LA/MA > 0.03 in the Italian cohort. All models were adjusted for age (per 5 years), waist circumference (per 5 cm), total cholesterol (per 10 mg/dL), diabetes, and hypertension.

As shown in Table 6, neither significant liver fibrosis (aOR 0.60; 95% CI 0.11–3.20; *p* = 0.55) nor elevated intestinal permeability (aOR 0.78; 95% CI 0.09–7.19; *p* = 0.83) was independently associated with intermediate–high ASCVD risk. In contrast, age remained the strongest determinant (aOR ≈ 4.7 per 5 years; *p* < 0.001), while other cardiometabolic factors showed modest, non-significant trends. Using a higher threshold (ASCVD ≥ 20%) yielded the same qualitative interpretation.

**Figure 1:**
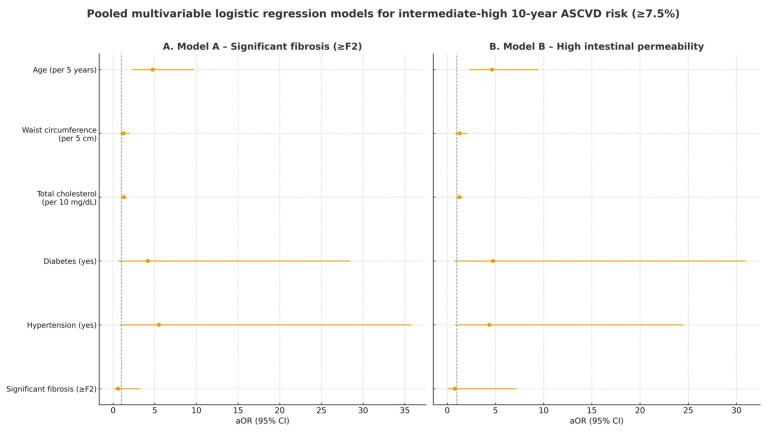
Forest plot of pooled multivariable logistic regression models evaluating predictors of intermediate-high (≥7.5%) 10-year ASCVD risk in 132 MASLD patients from the Romanian and Italian cohorts. (**A**) Model A includes significant liver fibrosis (≥F2) as exposure; (**B**) Model B includes high intestinal permeability as exposure. aOR: adjusted odds ratio; CI: confidence interval. The dotted vertical line represents the null effect value (OR = 1).

Figure 1 provides a visual summary of the adjusted odds ratios (aORs) and 95% confidence intervals for predictors of ASCVD ≥ 7.5% in the pooled dataset, complementing the tabular presentation in Table 6.

## 4. Discussion

### 4.1. Principal Findings

This study prospectively enrolled two independent European cohorts, MASLD cohorts, and mirrored real-world practice by using each center’s validated tools for fibrosis and gut–liver assessment. While assessing the specific cardiovascular risk, in both settings, the finding shows that most of participants clustered in the low or intermediate 10-year ASCVD risk categories (Table 2).

As expected, age and major cardiometabolic comorbidities—especially hypertension and diabetes—showed the most consistent associations with elevated ASCVD (≥7.5%), while other metabolic traits (adiposity, lipids, fasting glucose) displayed weaker, cohort-specific signals (Table 5A,B). Because events were limited, analyses were intentionally univariate with targeted age-adjusted checks and should be interpreted as exploratory. Given the exploratory purpose of the study, no multiple-comparison correction was applied; results should therefore be interpreted cautiously, with emphasis on effect sizes and consistency across cohorts rather than isolated *p*-values.

The interpretation of pooled analyses requires caution, as fibrosis and intestinal permeability were measured through different techniques in the two cohorts. While FibroMax and ARFI elastography are both validated methods for non-invasive fibrosis assessment, they rely on distinct physiological principles and yield non-equivalent absolute values. Similarly, fecal zonulin and serum inflammatory/permeability markers provide complementary but not directly comparable insights into gut barrier function. For this reason, pooled models were treated as exploratory and intended to highlight broader associations rather than to establish definitive causal pathways.

Overall, these results support the concept that cardiometabolic burden predominates as the driver of cardiovascular risk in subjects with MASLD characterized by a low-moderate ASCVD and in the absence of advanced liver fibrosis [28,29]. Of note, these results were confirmed in both cohorts, irrespective of the technique used to assess intestinal permeability and the grade of liver fibrosis.

### 4.2. Comparison with Previous Studies

Our data align with reports showing that cardiometabolic burden is the dominant determinant of cardiovascular risk in MASLD [30]. Consistent with this literature, hypertension and diabetes emerged as the most salient clinical correlates of elevated risk, echoing large population-based analyses linking these comorbidities to hepatic and cardiovascular morbidity [31,32,33]. The strong age gradient that we observed is likewise concordant with prior work, underscoring earlier identification and prevention in metabolically vulnerable adults [34,35].

By contrast, we did not observe a reproducible, independent signal for liver fibrosis with respect to higher 10-year risk. This differs from studies in which advanced fibrosis tracked incident cardiovascular events [36,37,38]. This discrepancy, however, seems to be mainly secondary to the small number of subjects with advanced liver fibrosis enrolled in both explored cohorts. Furthermore, our outcome predicted 10-year ASCVD risk rather than hard events, but several features may explain the divergence: our outcome was predicted 10-year ASCVD risk rather than hard events.

These considerations are compatible with the view that fibrosis is a robust hepatic prognostic marker, while its cardiovascular impact may operate largely through shared metabolic pathways rather than fibrosis per se [39].

Similarly, although mechanistic and translational data support a role for gut-barrier dysfunction in vascular injury [40,41] clinical associations with cardiovascular risk have been heterogeneous [42]. Using two different techniques (i.e., fecal zonulin or the lactulose-mannitol ratio), our analyses did not identify consistent associations between intestinal permeability and ASCVD categories, a pattern that fits the mixed evidence base and suggests any contribution of permeability to cardiovascular risk in MASLD is likely modest or non-independent in routine clinical settings.

### 4.3. Gut–Liver Axis and Regional Variations

This study deliberately analyzed gut–liver and fibrosis markers within each cohort because centers employed different, locally validated methods. The Romanian site used serum-based FibroTest and fecal zonulin, whereas the Italian site implemented ARFI elastography and a lactulose–mannitol absorption test; such heterogeneity reflects real-world practice and cautions against head-to-head comparisons [15,43,44].

The differing proportions of “increased intestinal permeability” observed within cohort likely mirror what each assay captures: zonulin reflects tight-junction regulation at the mucosal interface, while the LA/MA ratio represents a functional, primarily paracellular small-intestinal permeability readout [45,46,47]. These tests are complementary rather than interchangeable, and methodological factors—cut-offs, sample handling, and analytic platforms—can affect apparent prevalence and effect sizes [48]. Accordingly, results were reported descriptively and without direct cross-cohort statistical testing to avoid misleading inference.

Beyond methodological issues, lifestyle context may also differ by setting. Southern European populations, such as those in Italy, often show higher adherence to the Mediterranean diet and more favorable metabolic profiles, whereas Eastern European populations report lower adherence and a higher burden of obesity and metabolic syndrome [49,50]. These factors may partly shape baseline cardiometabolic risk and support the inclusion of standardized lifestyle assessments in future multi-country studies [35,50].

### 4.4. Clinical Implications

The non-trivial share of MASLD patients falling into the intermediate or high 10-year ASCVD categories has direct relevance for care pathways [51]. These individuals should be prioritized for early, comprehensive cardiometabolic management and for early assessment of the grade of liver fibrosis using standardized techniques. Results from the present study underscore the relevance of an aggressive control of hypertension and diabetes, of lifestyle optimization (dietary quality, weight reduction, physical activity), and evidence-based lipid-lowering in patients with MASLD [52,53].

In practice, ASCVD stratification can be used to tailor intervention intensity (e.g., thresholds for antihypertensive therapy, glycemic targets, and statin initiation) while hepatic evaluation continues in parallel to address liver-specific risks. Taken together, these results support a tiered approach: first, treat cardiometabolic burden decisively; second, use liver and gut-barrier assessments to refine overall prognosis and to guide future, harmonized studies [54].

### 4.5. Strengths and Limitations

In this study, the cardiovascular risk was harmonized a priori with 10-year ASCVD categories; analyses were primarily within cohort, with one exploratory between-cohort comparison of the ASCVD category distribution. Robustness checks (ASCVD ≥ 20% threshold, age-adjusted two-parameter checks for gut–liver/fibrosis markers, and Spearman correlations with continuous ASCVD%) supported the main results. Standard operating procedures and internal quality assurance were applied across sampling and laboratory steps.

Limitations include moderate sample sizes, the scarce number of subjects with advanced liver fibrosis and few ASCVD events, which limited precision and precluded stable multivariable models; center-specific measurement heterogeneity that limited head-to-head comparisons beyond the ASCVD category distribution; a cross-sectional design; self-report for some lifestyle/medication variables; and analytic variability inherent to zonulin and sugar absorption tests. In addition, the two centers used different assays for fibrosis and intestinal permeability (FibroTest and fecal zonulin in Romania; ARFI elastography and LA/MA ratio in Italy), reflecting local clinical practice rather than methodological choice; therefore, no inter-assay calibration was feasible. In this context, a key limitation of our study lies in the heterogeneity of diagnostic tools used across the two cohorts. Although each assay is validated and appropriate in its local clinical setting, their intrinsic differences limit the direct comparability of absolute values. This methodological variability also contributed to our choice not to build center-specific multivariable models and to interpret pooled analyses as exploratory. All laboratory analyses were performed within the same general study period, but not in a single batch, which may introduce minor batch-related variability. Use of Pooled Cohort Equations rather than a European engine may affect absolute calibration, although relative risk ranking is likely preserved. The ^13^C-methacetin breath test was summarized descriptively and was not entered into risk models. These constraints may attenuate small effects but do not alter the central observation that age and major cardiometabolic comorbidities dominated ASCVD categorization, whereas fibrosis and intestinal permeability markers showed no independent signals within the cohort. In particular, only five ASCVD ≥ 20% events occurred overall (four in Romania and one in Italy), which likely yielded unstable odds ratio estimates and very wide confidence intervals in the pooled multivariable models. Accordingly, these pooled analyses should be interpreted as exploratory; nevertheless, the direction and lack of statistical significance for intestinal permeability and fibrosis were consistent with the within-cohort results. Overall, the present analyses were designed to support harmonization of cardiovascular risk assessment in MASLD across centers, rather than to establish causal relationships between gut–liver axis markers, liver fibrosis, and ASCVD.

### 4.6. Future Directions

Future work should prioritize harmonization across centers and longitudinal design. First, protocols for fibrosis and gut–liver axis assessment ought to be standardized; where feasible, dual-modality sub-studies (e.g., FibroTest plus ARFI; zonulin plus LA/MA) would allow method comparison and calibration. Second, larger, prospectively powered European cohorts should collect the full covariate set to compute both ASCVD and SCORE2, with pre-specified analysis plans that include internal/external validation, calibration (in-the-large and slope), and assessment of clinical utility. Third, longitudinal follow-up with repeated measurements of intestinal permeability and fibrosis is needed to test trajectories and their association with incident cardiovascular events using time-varying models. Fourth, interventional studies should evaluate whether intensifying management of hypertension and diabetes, alongside lifestyle strategies (e.g., cardiometabolic nutrition) and microbiome-targeted approaches, can modify intestinal permeability and improve vascular outcomes. Fifth, mechanistic work integrating multi-omics (microbiome, metabolomics) with mediation and causal-inference frameworks may clarify how much cardiovascular risk is explained by classical cardiometabolic factors versus gut–liver pathways. Finally, methodological priorities include prospective plans for missing-data handling, center effects, and sensitivity analyses, as well as a core outcome set, common data model, and biobanking to support reproducibility and data sharing.

## 5. Conclusions

Across the two MASLD centers, 10-year ASCVD categories were driven mainly by age and established cardiometabolic comorbidities. Neither fibrosis stage nor intestinal permeability provided meaningful additional cardiovascular risk stratification in this low-to-moderate risk population without advanced liver fibrosis, independent of the assessment technique.

## Figures and Tables

**Table 1:** Baseline characteristics by cohort.

Parameter	Romania (*n* = 52)	Italy (*n* = 80)	*p*-Value
Age (years)	41.0 [27.8–58.0]	45.5 [27.8–54.2]	0.920
Female sex, *n* (%)	30 (57.7%)	39 (48.1%)	0.293
Male sex, *n* (%)	22 (42.3%)	41 (50.6%)	0.293
BMI (kg/m^2^)	26.1 [22.9–31.5]	28.0 [22.7–32.2]	0.620
Waist circumference (cm)	98.5 [80.8–102.0]	100.0 [79.5–100.9]	0.696
Fasting glucose (mg/dL)	93.0 [87.0–99.8]	85.0 [76.0–93.0]	<0.001
Fasting insulin (µU/mL)	9.0 [7.0–13.2]	8.9 [5.7–16.9]	0.863
HOMA-IR	2.1 [1.4–3.2]	1.9 [1.2–3.9]	0.670
Total cholesterol (mg/dL)	157.0 [137.5–193.2]	187.0 [160.8–215.8]	<0.001
HDL cholesterol (mg/dL)	57.5 [48.8–66.2]	53.0 [46.0–66.2]	0.225
LDL cholesterol (mg/dL)	78.0 [68.8–87.5]	108.4 [83.5–135.7]	<0.001
Systolic blood pressure (mmHg)	126.0 [120.8–133.2]	120.0 [110.6–129.2]	<0.001
Diastolic blood pressure (mmHg)	69.0 [65.0–74.5]	77.0 [70.2–80.0]	<0.001
Current smoking, *n* (%)	4 (7.7%)	13 (16.0%)	0.191
Alcohol consumption, *n* (%)	0 (0.0%)	0 (0.0%)	1.00

*n* (%); percentages are within cohort. Romania (*n* = 52) and Italy (*n* = 80); denominators equal cohort totals for all variables. Exploratory between-cohort tests: Mann–Whitney U (continuous) and Fisher’s exact/Chi-square (categorical), two-sided α = 0.05; no multiplicity adjustment. In the Italian cohort, alcohol consumption was 0%. Abbreviations: BMI, body mass index; HOMA-IR, homeostasis model assessment of insulin resistance.

**Table 2:** ASCVD risk distribution by cohort.

ASCVD Risk Category	Romania, *n* (%)	Italy, *n* (%)	*p*
Low (<5%)	41 (78.8%)	64 (80.0%)	0.872
Borderline (5–7.4%)	3 (5.8%)	5 (6.2%)	0.910
Intermediate (7.5–19.9%)	4 (7.7%)	10 (12.5%)	0.381
High (≥20%)	4 (7.7%)	1 (1.2%)	0.058

Ten-year ASCVD risk estimated with the 2013 ACC/AHA Pooled Cohort Equations (White race coefficients; treated vs. untreated systolic BP modeled). Values are *n* (%). *p*-values compare Romania vs. Italy using Fisher’s exact test for each category; an overall distribution comparison uses the Chi-square test. Two-sided α = 0.05; no multiplicity adjustment.

**Table 3:** Liver fibrosis staging in the Romanian and Italian cohorts.

Romanian Cohort (*n* = 52)		
Fibrosis stage	*n*	%
F0	36	69.2
F0–F1	3	5.8
F1	6	11.5
F1–F2	4	7.7
F2	1	1.9
F3–F4	1	1.9
F4	1	1.9
Italian cohort (*n* = 80)		
Fibrosis stage	*n*	**%**
F0	42	52.5
F1	24	30.0
F2	8	10.0
F3	3	3.8
F4	3	3.8

Fibrosis assessed by FibroTest (Romania) and ARFI elastography (Italy). No cross-cohort testing.

**Table 4:** Intestinal permeability in the Romanian and Italian cohorts.

Romanian Cohort (*n* = 52)	
Fecal zonulin (ng/mL), median [IQR]	56.5 [37.5–107.3]
Elevated zonulin (>107 ng/mL), *n* (%)	14 (26.9%) *
Italian cohort (*n* = 80)	
LA/MA ratio, median [IQR]	0.014 [0.011–0.017]
Elevated LA/MA ratio (>0.03), *n* (%)	5 (6.3%) *

Romania *n* = 52; Italy *n* = 80. Predefined cut-offs: zonulin > 107 ng/mL; LA/MA > 0.03. * significantly different (*p* = 0.002) by Chi-squared Fisher’s Exact test.

**Table 5:** Univariate predictors of elevated ASCVD (≥7.5%) within cohorts ((**A**): Romanian cohort; (**B**): Italian cohort).

	(A) Romanian Cohort	(B) Italian Cohort
Predictor	OR (95% CI)	OR (95% CI)
Age (per 5 years)	1.65 (1.17–2.34)	2.21 (1.41–3.45)
Male sex (vs. female)	35.76 (1.93–662.97)	30.56 (1.74–535.72)
BMI (per 1 kg/m^2^)	1.11 (0.99–1.26)	0.98 (0.89–1.08)
Waist (per 5 cm)	1.13 (0.86–1.47)	1.38 (1.01–1.88)
Total cholesterol (per 10 mg/dL)	1.26 (1.01–1.56)	1.09 (0.91–1.30)
HDL cholesterol (per 5 mg/dL)	0.78 (0.53–1.14)	0.78 (0.61–1.00)
Fasting glucose (per 10 mg/dL)	0.99 (0.66–1.49)	1.47 (1.00–2.17)
Hypertension	21.00 (2.89–152.58)	2.44 (0.44–13.60)
Diabetes	5.00 (1.02–24.41)	2.00 (0.20–19.71)
Current smoking	1.95 (0.18–21.54)	1.11 (0.22–5.71)
Significant fibrosis (≥F2)	3.00 (0.24–37.67)	1.33 (0.26–6.94)
IP high (zonulin > 107 ng/mL)	1.80 (0.37–8.79)	-
IP high (LA/MA > 0.03)	-	NA

(**A**) Data are within-cohort univariate logistic regressions with outcome ASCVD ≥ 7.5% (intermediate/high) vs. <7.5%. Predictors scaled as indicated. Values are odds ratios (OR) with 95% confidence intervals (CI). No cross-cohort comparisons were performed. (**B**) Data are within-cohort univariate logistic regressions with outcome ASCVD ≥ 7.5% (intermediate/high) vs. <7.5%. Predictors scaled as indicated. Values are odds ratios (OR) with 95% confidence intervals (CI). No cross-cohort comparisons were performed. For LA/MA > 0.03, no elevated-risk events occurred among positives; OR not estimable; Fisher’s exact *p* = 1.00.

**Table 6:** Pooled multivariable logistic regression models for intermediate-high 10-year ASCVD risk (≥7.5%) in 132 MASLD patients from the Romanian and Italian cohorts (Model A: significant liver fibrosis; Model B: high intestinal permeability).

Model A—Exposure: Significant Liver Fibrosis (≥F2)			
Variable	aOR	95% CI	*p*-value
Significant fibrosis (≥F2)	0.60	0.11–3.20	0.550
Age (per 5 years)	4.75	2.32–9.73	<0.001
Waist circumference (per 5 cm)	1.27	0.81–1.99	0.300
Total cholesterol (per 10 mg/dL)	1.28	0.99–1.64	0.060
Diabetes (yes)	4.16	0.61–28.41	0.150
Hypertension (yes)	5.50	0.85–35.77	0.070
Model B—Exposure: High intestinal permeability			
Variable	aOR	95% CI	*p*-value
High Intestinal Permeability	0.78	0.09–7.19	0.830
Age (per 5 years)	4.64	2.29–9.42	<0.001
Waist circumference (per 5 cm)	1.29	0.82–2.03	0.270
Total cholesterol (per 10 mg/dL)	1.26	0.98–1.62	0.070
Diabetes (yes)	4.75	0.73–30.92	0.100
Hypertension (yes)	4.37	0.78–24.48	0.090

Abbreviations: aOR = adjusted odds ratio; CI = confidence interval.

## Data Availability

The data presented in this study are available upon request from the corresponding author.

## Supplementary Materials

The following supporting information can be downloaded at: https://www.mdpi.com/article/10.3390/jcm14238361/s1, Table S1: Complete data distribution for 10-year ASCVD risk categories in the Romanian and Italian cohorts.

## Abbreviations

The following abbreviations are used in this manuscript:

MASLD	Metabolic dysfunction-associated steatotic liver disease
MASH	Metabolic dysfunction-associated steatohepatitis
NAFLD	Non-alcoholic fatty liver disease
NASH	Non-alcoholic steatohepatitis
CVD	Cardiovascular disease
BMI–	Body mass index
WC	Waist circumference
T2DM	Type 2 diabetes mellitus
HTN	Hypertension
TG	Triglycerides
HDL-C	High-density lipoprotein cholesterol
LDL-C	Low-density lipoprotein cholesterol
FPG	Fasting plasma glucose
HbA1c	Hemoglobin A1c
HOMA-IR	Homeostatic model assessment of insulin resistance
AST	Aspartate aminotransferase
ALT	Alanine aminotransferase
GGT	Gamma-glutamyl transferase
ALP	Alkaline phosphatase
TSH	Thyroid-stimulating hormone
ARFI	Acoustic radiation force impulse
ELISA	Enzyme-linked immunosorbent assay
PHQ-9	Patient Health Questionnaire-9
MET	Metabolic equivalents
OR	Odds ratio
CI	Confidence interval
SD	Standard deviation
IQR	Interquartile range
FibroTest	Fibrosis assessment test
SteatoTest	Steatosis assessment test
NashTest	Non-alcoholic steatohepatitis assessment test
