# Supplementary material for: Ten-Year Atherosclerotic Cardiovascular Disease Risk in Metabolic Dysfunction-Associated Steatotic Liver Disease (MASLD): Separate Analyses from Romanian and Italian Cohorts Integrating Metabolic, Hepatic, and Gut–Liver Axis Markers
Source: J Clin Med. 2025 Nov 25;14(23):8361. doi: 10.3390/jcm14238361 (PMC12693247; doi:10.3390/jcm14238361)
Supplement: Supplementary file 1 [file jcm-14-08361-s001.zip › jcm-3975914-supplementary.pdf]

Supplementary Table S1. Summary of raw descriptive data across fibrosis stages, ASCVD risk categories, and intestinal permeability in the Romanian (n = 52) and Italian (n = 80) cohorts. Values reproduced from Tables 1–4 of the main manuscript. Percentages are calculated within each cohort.

Table S1A. Liver Fibrosis Staging in Romanian and Italian Cohorts

Fibrosis Stage	Romania (n = 52)	Italy (n = 80)
F0	36 (69.2%)	42 (52.5%)
F0–F1	3 (5.8%)	—
F1	6 (11.5%)	24 (30.0%)
F1–F2	4 (7.7%)	—
F2	1 (1.9%)	8 (10.0%)
F3–F4	1 (1.9%)	—
F4	1 (1.9%)	3 (3.8%)

Assessment method: FibroTest (Romania), ARFI elastography (Italy).

Table S1B. Ten-Year ASCVD Risk Categories in Romanian and Italian Cohorts

ASCVD Risk Category	Romania (n = 52)	Italy (n = 80)
Low (<5%)	41 (78.8%)	64 (80.0%)
Borderline (5–7.4%)	3 (5.8%)	5 (6.2%)
Intermediate (7.5–19.9%)	4 (7.7%)	10 (12.5%)
High (≥20%)	4 (7.7%)	1 (1.2%)

Risk estimation: ACC/AHA Pooled Cohort Equations, White race coefficients.

Table S1C. Intestinal Permeability Measurements in Romanian and Italian Cohorts

Intestinal Permeability Marker	Romania (n = 52)	Italy (n = 80)
Median fecal zonulin (ng/mL)	56.5 [37.5–107.3]	—
Elevated zonulin (>107 ng/mL)	14 (26.9%)	—
Median LA/MA ratio	—	0.014 [0.011–0.017]
Elevated LA/MA ratio (>0.03)	—	5 (6.3%)
Cut-offs: zonulin >107 ng/mL; LA/MA >0.03.		
Methods: fecal zonulin ELISA (Romania); lactulose–mannitol HPLC test (Italy).		

Methods: fecal zonulin ELISA (Romania); lactulose–mannitol HPLC test (Italy).

Data are presented as median [IQR] or n (%). All values reflect raw, unadjusted cohort characteristics. No cross-cohort calibration was feasible due to methodological heterogeneity (FibroTest vs. ARFI; fecal zonulin vs. LA/MA ratio).
